# Protective effect of free phenolics from *Lycopus lucidus* Turcz. root on carbon tetrachloride-induced liver injury *in vivo* and *in vitro*

**DOI:** 10.29219/fnr.v62.1398

**Published:** 2018-07-25

**Authors:** Yue-Hong Lu, Cheng-Rui Tian, Chun-Yan Gao, Wen-Jing Wang, Wen-Yi Yang, Xiao Kong, You-Xia Chen, Zhen-Zhen Liu

**Affiliations:** 1 College of Food Engineering and Nutritional Science, Shaanxi Normal University, Xi’an, China; 2 College of Agronomy and Biological Science, Dali University, Dali, China; 3 School of Public Health, Dali University, Dali, China

**Keywords:** Lycopus lucidus Turcz, Free phenolics, Carbon tetrachloride, Hepatoprotective effect

## Abstract

Protective effect of free phenolics from *Lycopus lucidus* Turcz. root (FPLR) on CCl_4_-induced hepatotoxicity *in vivo* and *in vitro* was first evaluated. Oral administration of FPLR (100 mg/kg bw) to mice significantly reduced the CCl_4_-induced elevation of serum alanine aminotransferase, aspartate aminotransferase, alkaline phosphatase, triacylglycerols, total cholesterol, and total bilirubin. FPLR also increased the hepatic GSH contents and antioxidant enzyme activities of SOD and CAT and decreased the hepatic MDA level. Histopathological examinations further confirmed that the FPLR could protect the liver from CCl_4_-induced damage. Further research indicated that FPLR prevented the DNA fragmentation caused by CCl_4_ based on TUNEL assay. Moreover, immunohistochemistry staining demonstrated that pretreatment with FPLR significantly inhibited the elevation of hepatic TNF-α, IL-6, IL-8, iNOS, COX-2, and Caspase-3 in CCl_4_-treated mice. *In vitro* experiments showed that FPLR remarkably reduced BRL hepatocyte apoptosis and damage caused by CCl_4_ treatment. These findings indicate that FPLR could be developed as a functional food or medication for therapeutic purpose and prevention of hepatic injury.

**Figure:**
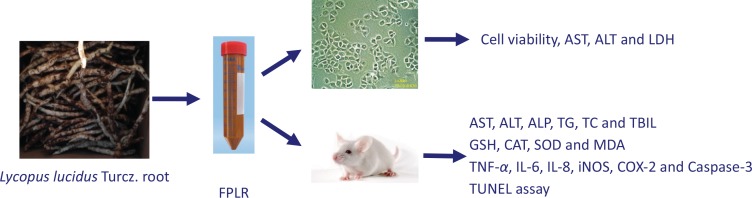

Liver plays a leading role in filtering and clearing blood received from digestive tract prior to passing it to other body tissues and organs. Furthermore, it is involved in detoxifying the body from hazardous substances, including xenobiotics and toxins, and in mediating drug transformations and metabolism. So, the liver is highly susceptible to damage from different toxins, viruses, and reactive oxygen and nitrogen species (1–2). Such damage is often associated with liver metabolic and synthetic dysfunctions which can result in many disorders, ranging from the transient elevation of levels of hepatic enzymes to life-threatening hepatic fibrosis, cirrhosis of the liver, and even hepatocellular carcinoma (3).

Nowadays, hepatic diseases aroused a severe health concern worldwide. Unfortunately, currently available pharmacological treatments are inadequate, and some synthetic medications can cause severe undesirable effects. Therefore, dietary and pharmacological interventions using novel herbal preparations have gained more interest for the amelioration of liver dysfunction (4). And recent studies aimed at characterizing the health-promoting properties of many natural plants and foods, especially those rich in phenolics known to possess remarkable antioxidant and hepatoprotective potencies (5–8).

*Lycopus lucidus* Turcz. (*L. lucidus*), an herbaceous perennial species of the Lamiaceae family, has been used as a traditional medicine in eastern Asian countries for the treatment of disorders of menstruation, amenorrhea, menstrual cramps, cardiovascular diseases, inflammation, edema, bellyache, traumatic injury, rheumatic arthritis, and thyroid (9). Many pharmacological activities of *L. lucidus* have been reported so far, such as antioxidant activity (10–12), anti-inflammation (13), anticancer and antimicrobial activity (14), immunological effect (15, 16), anti-allergic effect (17), hypoglycemic and hypolipidemic effect (18), as well as protection of renal damage (19).

The root, as an edible and medicinal part from *L. lucidus*, containing high nutritional value (12) and potential bioactivities, has recently become popular and been widely consumed in China as a typical plateau food. However, to our best knowledge, there is limited information about the root of *L. lucidus*. In our previous study, we found the free phenolic extract from *L. lucidus* root possessed high phenolic content and strong antioxidant activity, and consisted mainly of rosmarinic acid, rosmarinic acid methylester, and *N*-*trans*-feruloyl tyramine, with caffeic acid and 3, 4-dihy-droxyphenyl caffeate as minor compounds (20). In this study, we determine the hepatoprotective effect of the free phenolic extract from *L. lucidus* root on CCl_4_-induced liver injury *in vivo* and *in vitro*.

## Materials and methods

### Chemicals

The diagnostic kits for superoxide dismutase (SOD, NO. A001-1-1), glutathione (GSH, NO. A006-1), catalase (CAT, NO. A007-1-1), and malondialdehyde (MDA, NO. A003-1) were purchased from the Jiancheng Institute of Biotechnology (Nanjing, China). Rabbit anti-tumor necrosis factor-α (TNF-α), rabbit anti-interleukin-6 (IL-6), rabbit anti-interleukin-8 (IL-8), rabbit anti-cyclooxygenase-2 (COX-2), rabbit anti-caspase-3, rabbit anti-inducible nitric oxide synthase (iNOS) antibodies, and 3,3’-diaminobenzidine tetrahydrochloride (DAB) chromogenic agent were purchased from Servicebio Chemical Co. (Wuhan, China). Tunel kit (No.11684817910) was obtained from Roche Diagnostics (Germany). HRP goat anti-rabbit IgG was provided by KPL (USA). BRL hepatocyte was purchased from the Institutes for Biological Sciences Cell Resource Center (Shanghai, China). All other chemicals and reagents used in the experiments were of analytical grade.

### Plant material and extraction procedure

*L. Lucidus* roots were collected from Silian village with the annual average temperature at 15°C, the average annual rainfall of 600 mm, and a yellow brown soil (pH 5-6) in Jianchuan County (Yunnan, China; latitude, 26°53′ N; longitude, 99°90′ E; altitude, 2,200 m). Extraction of free phenolics was carried out by mixing 20 g of freeze-dried *L. Lucidus* root power with 400 mL of 80% methanol-water under an ultrasonic wave for 10 min at room temperature for three times. After centrifugation, the combined supernatants were concentrated to about 50 mL using a rotary vacuum evaporator. The aqueous suspension was adjusted at pH 2.0 using 6 M hydrochloric acid and extracted six times with ethyl acetate (30 mL each). The ethyl acetate extract was reduced until dried under vacuum at 35°C, and the resulting precipitate was dissolved in 60 mL of pure water. The phenolics were purified by X-5 macroporous resin. Briefly, 10 g of the pretreated resin was added to the phenolic solution and continually shaken using a water-bath shaker at 120 r/min and 25°C for 24 h. The resin was then first washed twice with ultrapure water and then desorbed with 100 mL of 70% ethanol at 120 r/min and 25°C for 24 h. Desorption solution was evaporated at 35°C under vacuum, and the resulting precipitate was freeze-dried to obtain dry extracts.

### Determination of phenolic content

The phenolic content of the free phenolic extract was determined according to the Folin–Ciocalteu colorimetric method as described by Lu et al. (12). Phenolic content was expressed as milligram of gallic acid equivalent per gram of extract (mg GAE/g). The phenolic content of the free phenolics from *Lycopus lucidus* Turcz. root (FPLR) was 567.29 mg GAE/g extract.

### Determination of hepatoprotective effect in vivo

#### Animals and experimental design

A total of 60 kunming male mice (body weight 18–22 g) were obtained from the Laboratory Animal Centre of Dali University [license number of the experimental animals: SCXK (Xiang 2013-0004)]. All animal procedures were conducted in strict conformation with the guidelines of Chinese Council for Animal Care. The animals were allowed to adapt to the environment for 1 week. Then, the mice were randomly divided into six groups (10 animals in each group). The animals were housed in an animal facility at 22 ± 1°C with a 12 h light–dark cycle, controlled humidity (50–60%) and air circulation, and fed a standard pelleted diet. The normal control and model groups (CCl_4_-treated) were given normal saline daily. Based on the results of the preliminary experiment, the low-dosage, medium-dosage, and high-dosage FPLR-treated groups were supplemented with FPLR at 50, 100, and 200 mg/kg bw·d for 28 days. Bifendate is a commonly used medication in the treatment of viral hepatitis and drug-induced liver injury; therefore, the positive control group was given bifendate (100 mg/kg bw·d) orally for 28 days. On the 29th day, all the groups except the normal group received 1% CCl_4_ (5 mL/kg bw, dissolved in rapeseed oil) after 16 h of administration of the FPLR and bifendate. All mice were administrated by gavage. Twenty-four hours after receiving of the CCl_4_, they were killed. Serum was separated by centrifugation at 3,000 rpm for 10 min and then stored at −20°C until analysis. Livers were dissected out from each animal and washed immediately with ice-cold saline to remove as much blood as possible, and stored at −40°C until further analysis. The liver and spleen index of every mouse was calculated according to the records of the body weight and corresponding liver and spleen weights using the following formula: Liver index = liver weight (mg)/body weight (g), Spleen index = spleen weight (mg)/body weight (g).

#### Determination of serum alanine aminotransferase (ALT), aspartate aminotransferase (AST), alkaline phosphatase (ALP), total cholesterol (TC), triacylglycerols (TG) and total bilirubin (TBIL)

Liver damage was assessed by estimating serum levels of ALT, AST, ALP, TG, TC, and TBIL using a clinical automatic biochemical analyzer (Hitachi 7180, Japan) and the results were expressed as U/L, U/L, U/L, mmol/L, mmol/L, and μmol/L, respectively.

#### Determination of antioxidant makers in liver tissue

Liver homogenate 10.0% (w/v) was prepared with frozen normal saline and centrifuged at 3,000 rpm for 10 min. The homogenate supernatant was used for the measurement of SOD, CAT, GSH, and MDA. The levels of all of these indicators were determined by following the instructions on the commercial kits and the results were expressed as Units/mg protein (U/mg prot), U/mg prot, mg/g prot, and nmol/mg prot, respectively.

#### Histopathological assessment of liver damage

Samples of liver tissues were fixed in 10% neutral buffered formalin for 24 h. Specimens were dehydrated with ethanol solution and embedded in paraffin, sectioned at 5 μm and stained with hematoxylin–eosin for histopathological examination.

#### TUNEL assay

TUNEL assay was performed on paraffin-embedded sections from control, model, and FPLR (100 mg/kg bw) pretreatment groups according to the protocol of the kit. Briefly, the dewaxed course was done as described below. After the incubation with Proteinase K solution for 20 min, the mixed solution of TdT and dUTP (1:9, v/v) was added on the sections and incubated at 37°C for 2 h. Then the sections were washed by PBS (pH 7.4) and placed in 3% H_2_O_2_ for 15 min in the dark to block endogenous peroxidase activity. Subsequently, 50 μL converter-POD was added on the samples and reacted at 37°C for 30 min. After that, the sections were washed and stained with the DAB solution and hematoxylin. Images were obtained using a light microscopy (40×). Ten microscopic fields in each group were randomly selected for the count of positive cells.

#### Immunohistochemical analysis for TNF-α, IL-6, IL-8, iNOS, COX-2, and Caspase-3

The paraffin sections were deparaffinized with xylene (three times, each for 15 min) and rehydrated with different concentration gradient of alcohol (100, 90, 70, and 60%) once for 5 min. Subsequently, the paraffin sections were treated with EDTA (pH = 8.0) in a microwave oven for 15 min for antigen retrieval. 3% H_2_O_2_ was used to block endogenous peroxidase activity for 25 min at room temperature and nonspecific protein binding was blocked by 3% bovine serum (BSA) for 30 min. The treated slides were incubated in a moist box at 4°C overnight with rabbit anti-TNF-α (1:200), rabbit anti-IL-6 (1:800), rabbit anti-IL-8 (1:100), rabbit anti-COX-2 (1:800), rabbit anti-caspase-3 (1:400), and anti-iNOS antibodies (1:1,000), followed by incubation in HRP-labeled goat anti-rabbit IgG for 50 min. DAB fluid including 1 mL distilled water, 50 μL H_2_O_2_ (20×), and 50 μL DAB (20×) was used in color development, and counterstained by hematoxylin. Image was taken by a light microscopy (40×).

### Determination of hepatoprotective effect in vitro

#### Determination of cell viability

BRL hepatocytes were seeded into 96-well plates at 2.5×10^4^/mL, and 100 μL were added in each well. Following 12 h of incubation, 100 μL of different concentrations of FPLR (1.6, 0.8, 0.4, 0.2 mg/mL, in DMEM containing 0.1% dimethyl sulfoxide) were added. The control and CC1_4_-treated groups were given 100 μL of DMEM. Then, after 4 h of incubation, 40 μL PBS were added to the control group, while the other groups were given 40 μL CC1_4_ (100 mM) to induce cell injury. Three hours later, cell viability was determined by microculture tetrazolium assay and the results were expressed as percent cell viability.

#### Determination of the AST, ALT and LDH activities in supernatants

BRL hepatocytes were seeded into 24-well plates at 2.5×10^4^/mL, and 500 μL were added in each well. Following 12 h of incubation, 500 μL of different concentrations of FPLR (1.6, 0.8, 0.4, 0.2 mg/mL, in DMEM containing 0.1% dimethyl sulfoxide) were added. The control and CC1_4_-treated groups were given 500 μL of DMEM. Then, after 4 h of incubation, 200 μL PBS were added to the control group, while the other groups were given 200 μL CC1_4_ (100 mM) to induce cell injury. Three hours later, the culture supernatants were harvested and the AST, ALT, and LDH activities in supernatants were determined using a clinical automatic biochemical analyzer (Hitachi 7180, Japan).

### Statistical analysis

All the experiments were performed in triplicates and the experimental data were expressed as mean ± standard deviation (SD). One-way analysis of variance (ANOVA) and Duncan’s multiple range test were conducted to determine significant differences between the means by SPSS (version 17.0).

## Results and discussion

### Hepatoprotective effect of FPLR in vivo

#### Effects of FPLR on liver and spleen index

The effects of FPLR on the liver and spleen index are shown in Table 1. It was observed that CCl_4_-exposed mice showed a significant increase (*p* < 0.05) in liver and spleen index compared with the normal control group, indicating that the liver and spleen tissues were severely damaged induced by CCl_4_. However, mice received bifendate (100 mg/kg bw) and the dose of FPLR (100 or 200 mg/kg bw) for 28 days showed a marked decrease (*p* < 0.05) in liver index in comparison with the CCl_4_-treated group, suggesting that the administration of bifendate and the tested FPLR resulted in a preventive effect against CCl_4_-induced liver swelling in mice. And the spleen index of the bifendate and FPLR pretreated groups was lower than that of CCl_4_-treated group with no significant difference.

**Table 1:** Effects of pretreatment with FPLR on the liver and spleen index in CCl_4_-treated mice ($\bar{X}$ ± SD, *n* = 10)

Groups	Liver index	Spleen index
Normal control	49.95 ± 1.69^d^	3.51 ± 0.35^b^
CCl_4_-treated	60.42 ± 1.47^a^	4.01 ± 0.58^a^
Bifendate (100 mg/kg bw) + CCl_4_	53.98 ± 1.11^bc^	3.71 ± 0.23^ab^
FPLR (50 mg/kg bw) + CCl_4_	55.01 ± 2.36^b^	3.89 ± 0.36^a^
FPLR (100 mg/kg bw) + CCl_4_	54.04 ± 1.25^bc^	3.82 ± 0.24^ab^
FPLR (200 mg/kg bw) + CCl_4_	52.99 ± 1.66^c^	3.73 ± 0.31^ab^

Data are shown as mean values ± SD for the 10 mice in each group. Values within a column with different letters indicate significant difference among different groups at *p* < 0.05.

#### Effects of FPLR on serum ALT, AST, ALP, TG, TC, and TBIL

The levels of ALT, AST, ALP, TG, TC, and TBIL in the serum were investigated to assess liver injury. As shown in Table 2, the levels of serum ALT, AST, ALP, TG, TC, and TBIL were significantly increased by 8.56-, 3.41-, 0.29-, 0.20-, 1.22-, and 12.79-fold in CCl_4_-treated group compared with the normal control group. However, the increases in levels of ALT, AST, ALP, TG, TC, and TBIL were significantly decreased in mice pretreated with FPLR at 100 mg/kg bw. It was worth mentioning that FPLR prevented these elevations not in a dose-dependent manner, which suggested that there might be antagonistic or synergistic effect between the bioactive compounds in the phenolic extract within a certain concentration range. Similarly, the levels of serum ALT, AST, ALP, TG, TC, and TBIL of the mice in bifendate group also remained at remarkably low levels compared with those of CCl_4_-treated group. Mice treated with CCl_4_ developed significant hepatic damage evidenced by substantial increases in the levels of serum ALT, AST, ALP, TG, TC, and TBIL that are indicators of cellular leakage, loss of functional integrity of cell membrane, and decline of metabolic capacity in liver tissue (21). The results obtained in the present study showed that FPLR may prevent CCl_4_-exposed hepatic injury through the recovery of function and structure of hepatic cell.

**Table 2:** Effects of pretreatment with FPLR on the levels of serum ALT, AST, ALP, TG, TC, and TBIL in CCl_4_-treated mice ($\bar{X}$ ± SD, *n* = 10)

Groups	ALT(U/L)	AST (U/L)	ALP (U/L)	TG (mmol/L)	TC (mmol/L)	TBIL (μmol/L)
Normal control	117.20 ± 23.59^d^	361.50 ± 28.02^e^	85.75 ± 4.65^b^	1.18 ± 0.06^d^	1.16 ± 0.11^e^	0.47 ± 0.08^c^
CCl_4_ -treated	1120.56 ± 189.36^a^	1594.89 ± 112.28^a^	110.67 ± 13.42^a^	1.42 ± 0.09^a^	2.58 ± 0.24^a^	6.48 ± 1.01^a^
Bifendate (100 mg/kg bw) + CCl_4_	610.70 ± 92.67^c^	698.50 ± 100.5^d^	92.9 ± 10.9^ab^	1.22 ± 0.08^cd^	1.61 ± 0.12^d^	3.31 ± 0.57^b^
FPLR (50 mg/kg bw) + CCl_4_	987.32 ± 101.31^ab^	1230.55 ± 163.54^bc^	103.38 ± 9.38^a^	1.31 ± 0.05^b^	1.92 ± 0.15^bc^	5.76 ± 1.12^a^
FPLR (100 mg/kg bw) + CCl_4_	839.59 ± 118.52^b^	1026.18 ± 97.17^cd^	84.89 ± 8.25^b^	1.28 ± 0.05^bc^	1.78 ± 0.21^cd^	5.31 ± 0.65^a^
FPLR (200 mg/kg bw) + CCl_4_	1011.75 ± 103.92^ab^	1335.78 ± 98.66^b^	105.00 ± 3.61^a^	1.30 ± 0.08^bc^	2.12 ± 0.10^b^	5.53 ± 0.89^a^

Data are shown as mean values ± SD for the 10 mice in each group. Values within a column with different letters indicate significant difference among different groups at *p* < 0.05.

#### Effects of FPLR on antioxidant makers in liver tissue

The liver MDA content was significantly increased in the animals of CCl_4_-treated group compared to normal control group (Table 3). In contrast, FPLR pretreatment significantly prevented the elevation of MDA. Similarly, treatment with bifendate, the positive control, also exerted a good protective effect. As shown in Table 3, the levels of liver SOD, CAT, and GSH showed remarkable decreases in the mice treated with CCl_4_ compared with normal control group. However, the bifendate pretreatment significantly increased the levels of liver SOD, CAT, and GSH compared with those of the CCl_4_-treated group. Similarly, the FPLR pretreated animals also showed a significant improvement and the protective effect of the FPLR at the dose of 200 mg/kg bw was similar to bifendate.

**Table 3:** Effects of pretreatment with FPLR on the levels of liver SOD, CAT, GSH, and MDA in CCl_4_-treated mice ($\bar{X}$ ± SD, *n* = 10)

Groups	SOD (U/mg prot)	CAT (U/mg prot)	GSH (mg/g prot)	MDA (nmol/mg prot)
Normal control	59.84 ± 5.98^a^	38.55 ± 2.78^a^	4.65 ± 0.73^a^	1.92 ± 0.52^d^
CCl_4_-treated	45.11 ± 4.22^d^	20.84 ± 2.14^d^	1.97 ± 0.48^c^	3.99 ± 0.93^a^
Bifendate (100 mg/kg bw) + CCl_4_	55.04 ± 5.27^ab^	32.35 ± 2.55^b^	4.20 ± 0.45^ab^	2.47 ± 0.39^cd^
FPLR (50 mg/kg bw) + CCl_4_	48.11 ± 2.36^cd^	26.95 ± 1.36^c^	3.56 ± 0.73^b^	3.32 ± 0.64^b^
FPLR (100 mg/kg bw) + CCl_4_	51.14 ± 4.46^bcd^	28.89 ± 2.24^bc^	3.88 ± 0.79^b^	2.86 ± 0.78^bc^
FPLR (200 mg/kg bw) + CCl_4_	53.21 ± 3.66^bc^	31.92 ± 4.61^b^	4.08 ± 0.73^ab^	3.16 ± 0.51^b^

Data are shown as mean values ± SD for the 10 mice in each group. Values within a column with different letters indicate significant difference among different groups at *p* < 0.05.

The liver injury induced by CCl_4_ results from free radicals and peroxidation. CCl_4_ hepatotoxicity derives from reactive metabolic trichloromethyl radical (CCl_3_•) and proxy trichloromethyl radical (•OOCCl_3_). These free radicals attack and destroy polyunsaturated fatty acid, in particular those associated with phospholipids, in turn, leading to lipid peroxidation in the liver cells which plays a significant role in the pathogenesis of diseases (22). MDA is one of the end products of lipid peroxidation in the liver tissue and its content reflects the degree of lipid peroxidation. An increase in MDA levels in the liver suggested enhanced peroxidation, leading to tissue damage and failure of the antioxidant defense mechanisms to prevent the formation of excessive free radicals (23). An antioxidant defense system of antioxidant enzymes plays an important role in preventing the oxidative damage induced by CCl_4_ on the liver. SOD, CAT, and GSH constitute a mutually supportive team of antioxidant defense against oxidative damage. SOD catalyzes the dismutation of the superoxide anion radicals by combining it with protons to form hydrogen peroxide and oxygen (24). CAT decomposes hydrogen peroxide to water and oxygen and prevents the production of hydroxyl radicals. GSH makes a great contribution to the detoxification of the reactive toxic derivatives of CCl_4_ and that liver necrosis begins when the GSH stores are markedly depleted (25). However, these antioxidant enzymes could be easily inactivated by lipid peroxides or reactive oxygen species (ROS) in toxicity. Therefore, the change in antioxidant enzyme activities is related to the capacity of the liver to deal with oxidative stress during CCl_4_ poisoning.

Many studies reported that phenolic compounds from plants protect against CCl_4_-induced liver injury in mice by reducing lipid peroxidation and elevation of antioxidant enzyme activity (7, 26–29). Results of the present study revealed that the MDA content was significantly decreased and the levels of SOD, CAT, and GSH were significantly increased in the liver in response to FPLR treatment compared with CCl_4_-treated group mice, indicating that FPLR had the ability to protect the CCl_4_-induced liver oxidative damage through inhibition of lipid peroxidation and induction of antioxidant enzymes.

Nuclear factor erithroid 2-related factor 2 (Nrf2) plays an important role in the cellular antioxidant defense system against oxidative stress. The induction of many antioxidant enzymes, such as heme oxygenease-1 (HO-1), quinine oxidoreductase (NQO1), SOD, and CAT, are regulated by Nrf2 (30). Previous reports have shown that resveratrol and Acanthopanax senticosus Harms aqueous extracts could inhibit the chemical-induced oxidative stress by increasing of antioxidant enzyme activities via the induction of Nrf2 (31, 32). In the present study, the increase in GSH content, and SOD and CAT activities in FPLR treatment groups may be associated with the activation of Nrf2 pathway.

#### Histopathological assessment of liver damage

**Fig. 1:**
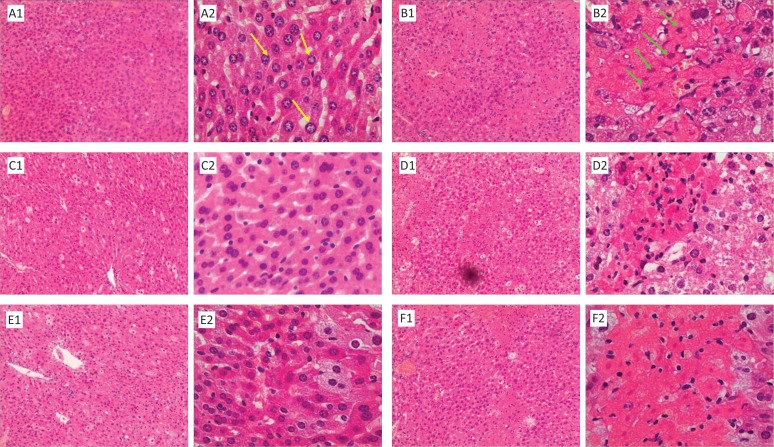
Images of histopathological examination (Hematoxylin and Eosin;1, 100×; 2, 400×). (A) normal control group; (B) treated with CCl_4_; (C) pretreated with bifendate (100 mg/kg bw), (D) pretreated with FPLR (50 mg/kg bw), (E) pretreated with FPLR (100 mg/kg bw) and (F) pretreated with FPLR (200 mg/kg bw) before CCl_4_ treatment. The yellow arrows indicate normal cellular architecture with a clear hepatic cell nucleus. The green arrows indicate hepatic cell necrosis.

Histopathological examination can provide the visual evidence for the hepatoprotective effects of investigated components against acute CCl_4_-induced liver injury. To further investigate the protective effect of FPLR on CCl_4_-induced liver injury in mice, the histopathological changes were assessed. In the normal group, liver slices showed typical hepatic cells with well-preserved cytoplasm, prominent nucleus and nucleolus, and visible central veins (Fig. 1A). In contrast, the liver sections of CCl_4_ treatment showed apparent histological changes including large areas of extensive hepatocyte necrosis, strong fatty changes of hepatocytes, condensed nuclei, massive inflammatory cells, and collapse of parenchyma (Fig. 1B). However, pretreatment with bifendate and FPLR at 100 mg/kg bw markedly ameliorated the liver damage caused by CCl_4_ (Fig. 1C and E). Results of histological examination were in good agreement with the liver functional parameters of the serum and hepatic antioxidant markers.

#### Effect of FPLR on hepatocyte apoptosis

**Fig. 2:**
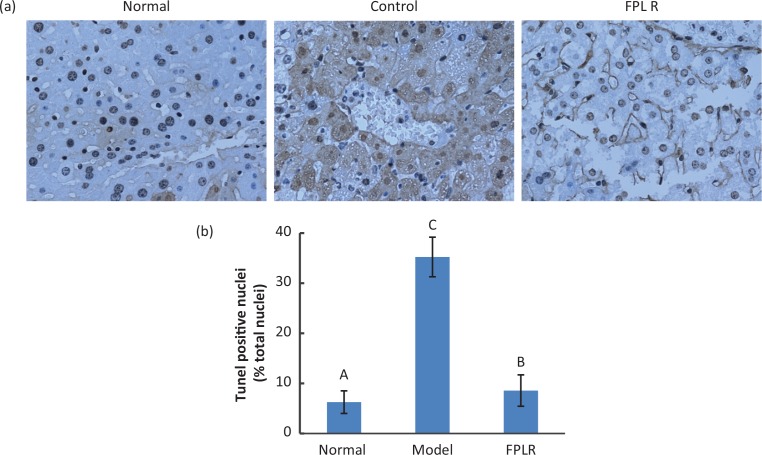
(a) TUNEL stained images (40×) in normal control, model and FPLR (100 mg/kg bw) + CCl_4_ groups. (b) Statistic analysis of TUNEL-stained images. Values expressed as mean ± SD in each group (*n* = 10). The different capital letters indicate significant differences among the different groups at *p* < 0.01.

To further investigate the effect of FPLR on hepatocyte apoptosis, the DNA fragmentation of nucleus was determined by TUNEL staining and the results were presented in Fig. 2. The nucleus of model group exhibited severe DNA fragmentation in comparison to normal control. However, the administration of FPLR (100 mg/kg bw) could obviously reduce the DNA fragmentation compared with model group (Fig. 2a and b). ROS produced by CCl_4_ is known to attack DNA and cause DNA fragmentation, in turn, leading to apoptosis. The results of the present study demonstrated that the FPLR had the potential to ameliorate the DNA damage, thus preventing the hepatocyte apoptosis induced by CCl_4_.

**Fig. 3:**
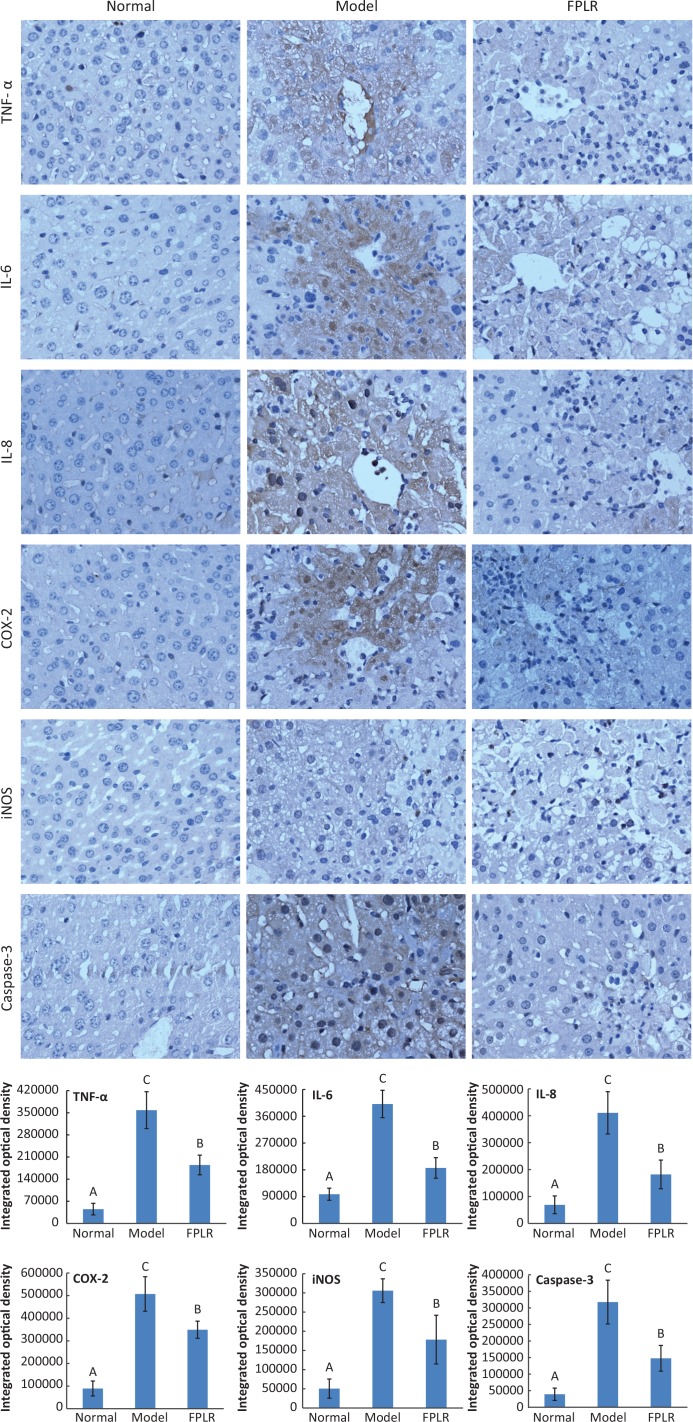
Effects of FPLR (100 mg/kg bw) on the levels of TNF-α, IL-6, IL-8, COX-2, iNOS, and Caspase-3 with the immunohistochemical method (40×). Statistical analysis was based on the values of integrated optical density. Values expressed as mean ± SD in each group (*n* = 10). The different capital letters indicate significant differences among the different groups at *p* < 0.01.

In addition, CCl_4_-intoxication has been suggested to cause severe apoptosis in the liver by activating Caspase-3, which is released to plasma by secondary necrosis. Our results showed that pretreatment with FPLR remarkably decreased the level of Caspase-3 in CCl_4_-treated mice (Fig. 3), which indicated that FPLR could inhibit hepatocyte apoptosis triggered by CCl_4_.

#### Effects of FPLR on the levels of TNF-a, IL-6, IL-8, COX-2, and iNOS

Immunohistochemical analysis revealed that the levels of hepatic TNF-α, IL-6, IL-8, COX-2, and iNOS increased considerably after CCl_4_ administration with the exhibition of brown staining (Fig. 3). In contrast, FPLR (100 mg/kg bw) significantly attenuated the increases in the levels of these parameters (Fig. 3).

Free radicals induced by CCl_4_ activate the innate immune system and Kupffer cells by the NADPH oxidase pathway or intracellular ROS-dependent kinase activation (28). Activated Kupffer cells release proinflammatory cytokines and activate other nonparenchymal cells involved in liver inflammation. TNF-α is produced by resident macrophages in CCl_4_-exposed liver tissue and subsequently stimulates the release of cytokines from macrophages and induces oxidative metabolism of phagocyte and the production of nitric oxide (33). Excessive nitric oxide produced by iNOS can react with ROS to form peroxynitrite radicals with strong cytotoxicity, which inhibits the synthesis of hepatocyte proteins to cause the disorder of hepatocyte metabolism, resulting in hepatocyte apoptosis and necrosis (27). In addition, TNF-α can also cause the occurrence of many secondary mediators in inflammatory response, such as IL-6, IL-8, and IL-1β. COX-2 is also an important mediator of inflammation, which is induced by proinflammatory cytokines, leading to formation of proinflammatory substrates from arachidonic acid (28). Our results showed that FPLR (100 mg/kg bw) significantly inhibited the elevation of hepatic TNF-α, IL-6, IL-8, iNOS, and COX-2 in CCl_4_-treated mice, which indicated that FPLR might suppress the inflammatory response by inhibiting the inflammatory cytokines.

In the course of inflammation responses, TNF-α is initiated by TNF-α receptor 1 (TNFR1), and its activation by CCl_4_ causes activation of nuclear factor kappa B (NF-κB), mitogen-activated protein (MAP) kinases, and activator protein 1 (AP-1). NF-κB, as a transcription factor, regulates expression of several genes related to infection and inflammation. The MAP kinase family regulates cell growth, cell death, stress adaptation to the environment, and inflammatory response in many important cellular pathological processes. AP-1 participates in cell proliferation and differentiation, is sensitive to ROS, as well as to lipid peroxidation end products (29). In addition, toll-like receptors (TLRs), a member of the pattern recognition receptor family, play an important role in mediating infection and inflammation under pathological conditions (28). Previous studies have shown that NF-κB, MAP kinase, TLRs, and AP-1 signaling pathways are involved in the pathophysiology of acute liver damage (26–29). Zhang et al. indicated that pretreatment with the flavonoids from *R. laevigata* Michx fruit dramatically prevented CCl_4_-induced liver damage in mice by decreasing the expressions of TNF-α, iNOS, and NF-κB (26). Niu et al. revealed that fraxin downregulated the TNF-α, IL-6, COX-2, iNOS, and nitric oxide production by inhibition of NF-κB and MAP kinases activation in CCl_4_-treated mice and HepG2 cells (27). In addition, Kim et al. reported that ferulic acid protects against CCl_4_-induced liver damage in mice through decreasing of TNF-α, iNOS, COX-2, and TLR4 expression, and inhibiting of JNK and p38 MAP kinases activation and AP-1 and NF-κB transactivation (28). Ko et al. also demonstrated that *Rhus verniciflua* Stokes glycoprotein (36 kDa) decreased the NF-κB and AP-1 activation in CCl_4_-treated mice (29). Therefore, the protective effect of FPLR on liver damage induced by CCl_4_ may be associated with the inflammatory signaling transduction pathway of NF-κB, MAP kinase, TLRs, and AP-1.

### Hepatoprotective effect of FPLR in vitro

In the present study, the BRL hepatocyte was injured by CCl_4_. Cell viability was used as an indicator to evaluate the protective effects of FPLR on the BRL hepatocyte injury induced by CCl_4_. The effects of pretreatment with FPLR on CCl_4_-induced alteration in cell viability of BRL hepatocyte are presented in Table 4. The cell viability in the CCl_4_-treated group was significantly decreased compared with the normal control group, while there was a dramatic increase in cell viability in the FPLR-treated groups in comparison with the CCl_4_-treated group.

In addition, CCl_4_-induced liver damage results in leakages of ALT, AST, and LDH from the cells. Therefore, the ALT, AST, and LDH activities in the culture supernatants were determined to comprehensively evaluate the hepatoprotective effects of the FPLR. Comparing with the control group, ALT, AST, and LDH activities were significantly increased by 98.16, 292.61, and 112.77% in supernatants in the CCl_4_-treated group, respectively. Marked increased release of ALT, AST, and LDH indicates severe damage to the cell membranes during CCl_4_-induced hepatocyte damage. However, pretreatment with FPLR significantly prevented the elevation of ALT, AST, and LDH activities in supernatants induced by CCl_4_. Furthermore, a dose-dependent manner was observed between FPLR concentrations and cell viability, as well as ALT, AST, and LDH activities. These results indicated that the free phenolic extract from *L. lucidus* roots could reduce BRL hepatocyte apoptosis and damage induced by CCl_4_, which further confirmed that the FPLR could protect the liver from CCl_4_-induced damage.

**Table 4:** Effects of pretreatment with FPLR on cell viability and AST, ALT, and LDH activities of BRL hepatocyte injured by CC1_4_ ($\bar{X}$ ± SD, *n* = 5)

Groups	Cell viability (%)	ALT (U/L)	AST (U/L)	LDH (U/L)
Normal control	92.66 ± 0.29^f^	1.63 ± 0.15^a^	4.33 ± 0.58^a^	75.67 ± 2.08^a^
CCl_4_-treated	23.64 ± 1.34^a^	3.23 ± 0.21^d^	17.00 ± 1.73^d^	161.00 ± 3.00^e^
FPLR (0.2 mg/mL) + CCl_4_	45.04 ± 2.91^b^	2.87 ± 0.21^cd^	15.67 ± 1.15^cd^	148.00 ± 1.73^d^
FPLR (0.4 mg/mL) + CCl_4_	57.33 ± 2.09^c^	2.47 ± 0.12^bc^	13.67 ± 0.58^bc^	143.33 ± 1.53^d^
FPLR (0.8 mg/mL) + CCl_4_	64.59 ± 0.77^d^	2.10 ± 0.20^ab^	12.67 ± 1.53^bc^	133.67 ± 2.52^c^
FPLR (1.6 mg/mL) + CCl4	70.39 ± 1.77^e^	1.73 ± 0.21^a^	12.00 ± 1.00^b^	117.67 ± 0.58^b^

Data are shown as mean values ± SD (n = 5). Values within a column with different letters indicate significant difference among different groups at *p* < 0.05.

Previous researches have demonstrated that *L. lucidus* possessed significant antioxidative and anti-inflammatory activities (10–13). Moreover, rosmarinic acid and caffeic acid had a potency of hepatic protection (34, 35). Therefore, it is reasonable to suggest that the protective potency of FPLR on CCl_4_-induced liver damage was due to the presence of bioactive phytochemicals in FPLR, especially rosmarinic acid and caffeic acid, which might contribute to hepatoprotective effects individually or collectively.

## Conclusions

In summary, the results obtained from *in vivo* experiment showed pretreatment with FPLR remarkably decreased serum ALT, AST, ALP, TG, TC, and TBIL levels, hepatic MDA content and DNA fragmentation, and improved pathological change by increasing GSH content and activities of SOD, CAT and attenuating of inflammatory cytokines and Caspase-3 expression in CCl_4_-intoxicated mice, which suggested that FPLR exerts a protective effect against CCl_4_-induced liver injury through activating the antioxidant system and suppressing inflammatory response. Moreover, results obtained from an *in vitro* experiment also revealed that FPLR could reduce BRL hepatocyte apoptosis and damage induced by CCl_4_ exposure. The hepatoprotective activity of FPLR might be associated with rosmarinic acid and caffeic acid. This is the first report on the hepatoprotective effects of phenolic extract from *L. lucidus* root, which provided evidence for medicinal and functional food uses of this plant. Further research is still imperative to elucidate the hepatoprotective mechanism of FPLR in CCl_4_-induced hepatic injury mice and hepatocyte.
